# Vaccination directed against the human endogenous retrovirus-K envelope protein shows efficiency in a murine tumor model system

**DOI:** 10.1186/1742-4690-10-S1-P81

**Published:** 2013-09-19

**Authors:** Benjamin Kraus, Katrin Fischer, Sarah Büchner, Katja Sliva, Barbara Schnierle

**Affiliations:** 1 Paul-Ehrlich-Institut, Dep. Virology, Langen, Germany

## 

Human endogenous retrovirus (HERV) genomes are chromosomally integrated in all cells of an individual. They are normally transcriptionally silenced and transmitted only vertically. Enhanced expression of HERV-K accompanied by the emergence of anti-HERV-K-directed immune responses has been observed in tumor patients and HIV-infected individuals. As HERV-K is usually not expressed and immunological tolerance development is unlikely, it is an appropriate target for the development of immunotherapies of the HIV infection. We generated a recombinant vaccinia virus (MVA-HKenv) expressing the HERV-K envelope glycoprotein (Env), based on the modified vaccinia virus Ankara (MVA), and established an animal model to test its vaccination efficacy. Murine renal carcinoma cells (Renca) were genetically altered to express *E. coli* beta-galactosidase (RLZ cells) and the HERV-K *Env* gene (RLZ-HKenv cells). Intravenous injection of RLZ-HKenv cells into syngenic BALB/c mice led to the formation of pulmonary metastases, which were detectable by X-gal staining. A single vaccination of tumor-bearing mice with MVA-HKenv drastically reduced the number of pulmonary RLZ-HKenv tumor nodules compared to vaccination with wild-type MVA. Prophylactic vaccination of mice with MVA-HKenv precluded the formation of RLZ-HKenv tumor nodules, whereas wild-type MVA-vaccinated animals succumbed to metastasis. Protection from tumor formation correlated with enhanced HERV-K Env-specific killing activity of splenocytes. These data demonstrate for the first time that HERV-K Env is a useful target for vaccine development and might offer new opportunities as an HIV vaccine.

